# Diagnostic value of contrast-enhanced ultrasound combined with MRI for cervical hyperplastic, tuberculosis-infected, and metastatic lymph nodes

**DOI:** 10.12669/pjms.39.4.7572

**Published:** 2023

**Authors:** Xiulei Yu, Wenzhi Zhang, Ning He, Dongming Su, Yang Zhao

**Affiliations:** 1 Xiulei Yu, Department of Ultrasonography, Hangzhou Chest Hospital, Zhejiang University School of Medicine, Hangzhou 310003, Zhejiang Province, P.R. China; 2 Wenzhi Zhang Department of Ultrasonography, Hangzhou Chest Hospital, Zhejiang University School of Medicine, Hangzhou 310003, Zhejiang Province, P.R. China; 3 Ning He Department of Ultrasonography, Hangzhou Chest Hospital, Zhejiang University School of Medicine, Hangzhou 310003, Zhejiang Province, P.R. China; 4 Dongming Su Department of Ultrasonography, Hangzhou Chest Hospital, Zhejiang University School of Medicine, Hangzhou 310003, Zhejiang Province, P.R. China; 5 Yang Zhao Department of Radiology, Hangzhou Chest Hospital, Zhejiang University School of Medicine, Hangzhou 310003, Zhejiang Province, P.R. China

**Keywords:** Contrast-enhanced ultrasound, Magnetic resonance imaging, Hyperplastic lymph nodes, Lymph node tuberculosis, Metastatic lymph nodes

## Abstract

**Objective:**

To explore the diagnostic value of contrast-enhanced ultrasound (CEUS) combined with magnetic resonance imaging (MRI) for cervical abnormal lymph nodes.

**Methods:**

We retrospectively reviewed the clinical records of 150 patients undergoing lymph node examinations at Hangzhou Chest Hospital from January 2017 to December 2019. According to the characteristics of lymph nodes, the patients were divided into three groups: 45 patients had hyperplastic lymph nodes (HLNs; Group-A), 55 had lymph node tuberculosis (LNTB; Group-B), 50 had metastatic lymph nodes (MLN; Group-C). We compared the ultrasonic examination and MRI results between the groups, and compared the diagnostic value of CEUS alone and CEUS plus MRI.

**Results:**

Lower resistance indexes (RI) for Groups-A and B than Group-C(*P*<0.05). Mixed blood flow type was predominant in Group-A, while the lymphohilum type was predominant in Group-B, and the marginal type was predominant in Group-C(*P*<0.05). The proportion of non-uniform types in Group-B was significantly higher than that in Groups-A and C(*P*<0.05). After enhancement, the proportions of non-uniform types in Groups-A and B were higher than Group-C(*P*<0.05). The results of MRI examination showed that positive reinforcement integral (PEI) and maximum slope of increase (MSI) values increased sequentially from Group-B to Group-A, and then to Group-C(*P*<0.05); while time to peak (TTP) values increased sequentially from Group-C to Group-A, and then to Group-B(*P*<0.05). The diagnosis accuracy of CEUS combined with MRI was significantly higher than that of CEUS alone(*P*<0.05). RI-PEI, RI-MSI, and RI-TTP showed high specificity and sensitivity in the diagnosis and differentiation of HLNs, LNTB, and MLNs(*P*<0.05).

**Conclusion:**

CEUS combined with MRI can significantly facilitate the differential diagnosis between HLNs, LNTB, and MLNs. The two diagnosis methods combined improve the diagnosis accuracy of cervical lymph node diseases.

## INTRODUCTION

Lymph nodes (LNs) are essential immune system structures in the human body that filter lymph and are the sites of immune cells proliferation. LNs are usually less than 0.5 cm in diameter.^1^ Under specific conditions, the lymph nodes become antibody-production sites to resist pathogenic factors. During this process, the lymph nodes undergo reactive hyperplasia and swelling.^2^ Hyperplastic lymph nodes (HLNs), lymph node tuberculosis (LNTB), and metastatic lymph nodes (MLN) are among the most common cervical lymph node diseases.^3^,^4^

The severity of cervical lymph node diseases and their clinical treatment methods vary greatly, but early differential diagnoses of HLN, LNTB, MLN and other diseases are critical to determine treatment methods and prognoses.^2^,^3^ Clinical diagnosis of cervical lymph node diseases is mainly arrived at through puncture and surgical pathology or though imaging examinations.^3^,^5^ Because of the complex influencing factors of cervical LN enlargement, some patients cannot be diagnosed after puncture or surgical pathology examinations alone.^6^ With the development of imaging techniques, CEUS and MRI have been widely used for diagnosing various tumoral diseases.^7^,^8^ Many studies on the early diagnosis of HLN, LNTB, MLN and other diseases using CEUS exist, but few researchers have assessed the diagnostic values of MRI or CEUS combined with MRI for the same pathologies. Therefore, we reviewed the clinical data of 45 patients with HLN, 50 with LNTB, and 55 with MLN who underwent CEUS combined with MRI examinations in our hospital to assess the diagnostic value of the combined approach in differentiating cervical LN diseases.

## METHODS

**Fig.1:**
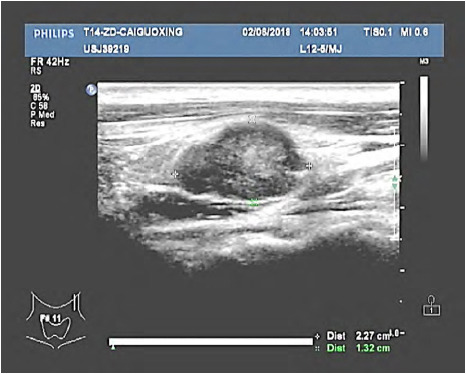
Multiple lymph nodes in the left neck were enlarged with unclear borders. Some lymph nodes were fused. The larger one was located in Zone III, with a size of about 2.6 × 1.1cm, the cortex is slightly hyperechoic, the internal echo is not uniform, the lymphatic hilum is clear, and the compression becomes narrow.

**Fig.2:**
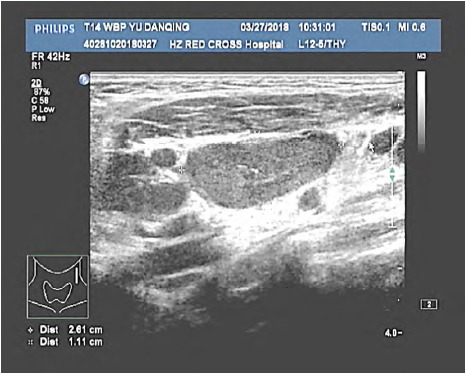
Multiple enlarged lymph nodes were detected in right cervical region II, and the larger ones were about 2.3×1.3cm, cortex thickened, internal echo was disordered and uneven, and lymphatic hilum disappeared.

**Fig.3:**
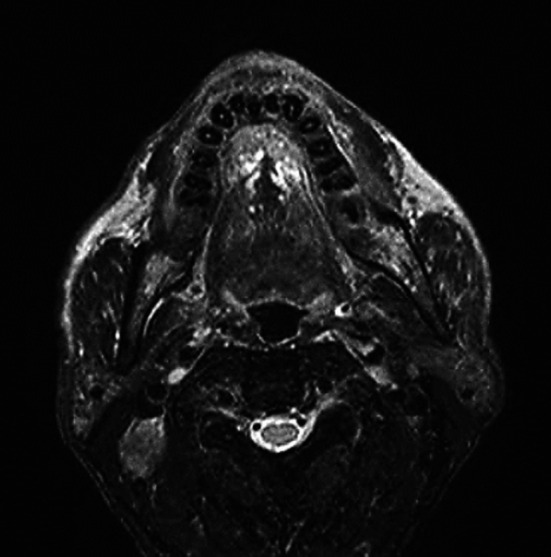
T2 transverse section right cervical lymph node metastasis, high signal intensity on T2WI.

We retrospectively reviewed the clinical records of 150 patients with LNs treated in Hangzhou Chest Hospital from 2017 to 2019. According to the nature of the LNs, 45 patients with HLN were assigned to Group-A (Fig.1), 55 with LNTB were assigned to Group-B (Fig.2), and 50 with MLN were assigned to Group-C (Fig.3). We found similar basic data among the three groups (*P*>0.05).

### Ethical Approval:

The Medical Ethics Committee at our institution approved the study (No 2021-16, Date: 2021-04-26). Informed consent was obtained from all patients.

### Inclusion criteria:

- Patients with diagnoses confirmed by histopathology results^9^
- Patients without allergies or other adverse reactions to the instruments and equipment used in this study
- Patients older than 18 years
- Patients with good nutritional and physical health levels without underlying diseases

### Exclusion criteria:

- Patients with congenital immune deficiency and/or serious infectious diseases
- Patients with severe heart, liver or kidney dysfunctions
- Patients with mental diseases or poor mental status
- Patients allergic to contrast media
- Patients with incomplete clinical data

### CEUS detection method:

**Fig.4:**
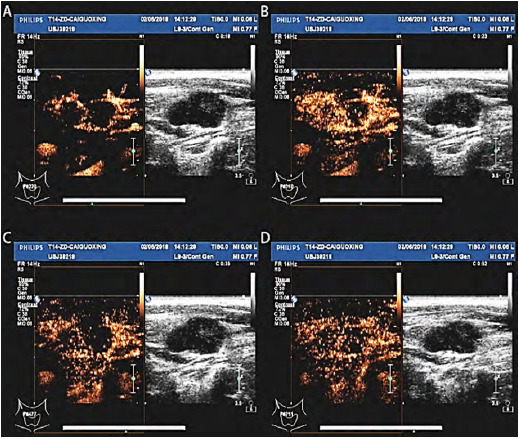
A to D: The capsule began to strengthen 16 seconds after the mass injection of ultrasound contrast agent. Enhancement peak after 23s with lymph nodes showing a centripetal pulsatile enhancement and absence of visible interior enhancement area. Clear lymph node outline at 35s and at 52s.

Philips iU22 was used as the color Doppler ultrasound diagnostic instrument with the probe frequencies at 3 to 9 MHz and 5 to 12 MHz. The patients were instructed to lie flat or on their side. First a routine ultrasound was performed to determine the number and location of lesions. Next, we applied a contrast mode (SonoVue; Italian Bracco Company) and set the mechanical index to 0.06–0.08. Before use, we dissolved the contrast agent dry powder in 5-ml of normal saline. We established an elbow vein channel, injected 2.4 ml of shadow making agent, and immediately flushed with 5-ml of normal saline. We observed the perfusion of contrast agent and adhered to the manufacturer’s instructions (Fig.4, A–D). Enhancement patterns were classified as homogeneous enhancement, septum-like enhancement, rim-like enhancement and no enhancement. Homogeneous enhancement was referred to simultaneous arrival of contrast agents in different parts of the same LN. Septum-like enhancement was defined as the internal rendering into a partition or honeycomb enhancement, while rim-like enhancement was defined as the edge and surrounding.

### MRI detection method:

We used the 3.0T superconducting magnetic resonance imaging system (Ingenia 3.0T all digital magnetic resonance system; Philips) to detect the PEI, MSI, TTP, and DTI of the patients in the three groups, and we strictly follow the instrument instructions.

All procedures above were performed by two radiologists with over five years of diagnostic experience who were blinded to the pathological results. In case of disagreement, it was negotiated with a third senior physician.

We obtained values for Vmax, resistance index (RI), blood flow type, enhancement mode, homogeneous enhancement type, and size change after CEUS enhancement; and the MRI results included PEI, MSI, TTP and DTI values. We generated a ROC curve to analyze the diagnostic value of related test indexes for HLN, LNTB, and MLN.

### Statistical analysis:

SPSS 25.0 software package was used for statistical analysis. Measurement data were expressed as means ± standard deviations (*χ̅*±*S*), with F-tests were used for inter Group-Analysis. The counting data were recorded as number (%), and Chi square tests were used to analyze the comparisons between the groups. ROC curve was used to analyze the diagnostic value of related test indexes for HLN, LNTB and MLN. All *P-*values <0.05 indicates the statistically significant differences. GradpadPrism7.0 was used for drawings.

## RESULTS

**Fig.5:**
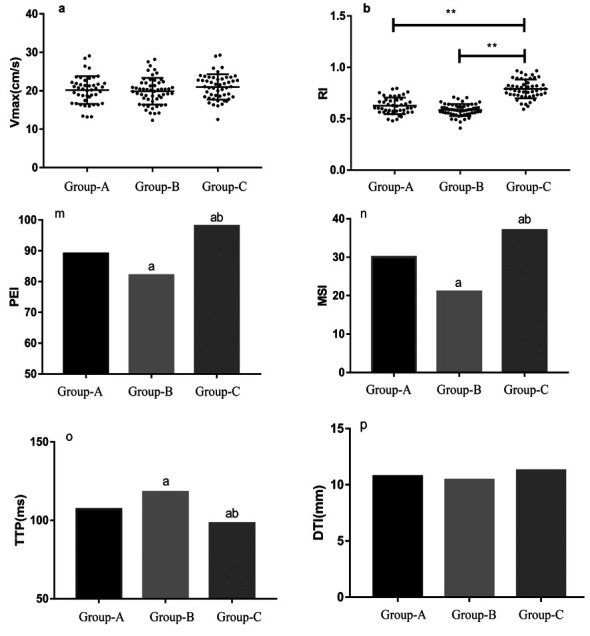
Comparison of A) ultrasonic examination results B) MRI results in the three groups.

***Note:*** ^a^ compared with Group-A, *P*<0.05; ^b^ compared with Group-B, *P*<0.05; ** is *P*<0.001

The CEUS examination results showed that the RI values of Group-A and Group-B were significantly smaller than those of Group-C (*P*<0.05), but the Vmax values were similar among the three groups (*P*>0.05) (Table-I). The blood flow types were mostly mixed for Group-A, lymphatic hilum for Group-B, and marginal for Group-C (*P*<0.05). The homogeneous enhancement proportions in Group-B were significantly higher than those in Group-A and Group-C (*P*<0.05). The proportions of abnormal lymph nodes increased after enhancement in Groups-A and B, but the proportion remained the same in Group-C (*P*<0.05). We found similar enhancement modes among the three groups (*P*>0.05; Table-II, and Fig.5A).

**Table-I:** Comparison of general data among the three groups.

Characteristics		Group-A (n=45)	Group-B (n=55)	Group-C (n=50)
Age (years)		45.37±5.24	47.48±5.62^a^	46.19±5.32^ab^
BMI (kg/m^2^)		21.49±1.13	22.36±1.15^a^	21.25±1.12^ab^
Gender(n%)	Male	25 (55.56)	33 (60.00) ^a^	27 (54.00) ^ab^
	Female	20 (44.44)	22 (40.00) ^a^	23 (56.00) ^ab^
Course of disease (year)		6.31±1.49	6.18±1.35^a^	6.53±1.61^ab^

***Note:***

a P<0.05, compared with Group-A; ^b^ P<0.05, compared with Group-B.

**Table-II:** Comparison of Ultrasonic examination and MRI results between the three groups.

Variables			Group-A (n=45)	Group-B (n=55)	Group-C (n=50)
Vmax (cm/s)			20.74±3.05	20.35±3.09	21.09±3.14
RI			0.62±0.07	0.59±0.06	0.78±0.09^ab^
Enhancement mode	Centripetal	21 (46.67%)		27 (49.09%)	27 (54.00%)
	Apocentricity	24 (53.33%)		28 (50.91%)	23 (46.00%)
Blood flow type	Marginal type	6 (13.33%)		10 (18.82%)	34 (68.00%) ^ab^
	Lymphatic hilum type	8 (17.78%) ^b^		24 (43.64%)	8 (16.00%) ^b^
	Mixed type	27 (60.00%)		4 (7.27%) ^a^	5 (10.00%) ^a^
	Central type	4 (8.89%)		7 (12.73%)	8 (16.00%)
Enhanced uniform type	Homogeneous type	18 (40.00%) ^b^		6 (10.91%)	20 (40.00%) ^b^
	Uneven type	25 (55.56%) ^b^		45 (81.82%)	27 (54.00%) ^b^
	Non reinforced	2 (4.44%)		4 (7.27%)	3 (6.00%)
Enhanced size	Enlarge	26 (57.78%)		29 (52.73%)	7 (14.00%) ^ab^
	Unchanged	19 (42.22%)		26 (47.27%)	43 (86.00%) ^ab^
PEI			89.53±8.32	82.47±7.24^a^	98.15±10.35^ab^
MSI			30.42±5.94	21.56±4.31^a^	37.29±7.63^ab^
TTP (ms)			107.26±14.37	118.39±16.48^a^	98.35±12.57^ab^
DTI (mm)			10.73±3.46	10.42±3.51	11.25±3.49

***Note:***

a P<0.05, compared with Group-A;

b P<0.05, compared with Group-B.

The MRI results showed that the PEI and MSI values increased sequentially from those in Group-B to those in Group-A, and to those in Group-C; and the TTP values increased from those in Group-C to those in Group-A, and to those in Group-B. The differences between the groups were statistically significant (*P*<0.05), while the DTI values of the three groups were similar (*P*>0.05; Table-II, and Fig.5B).

**Fig.6:**
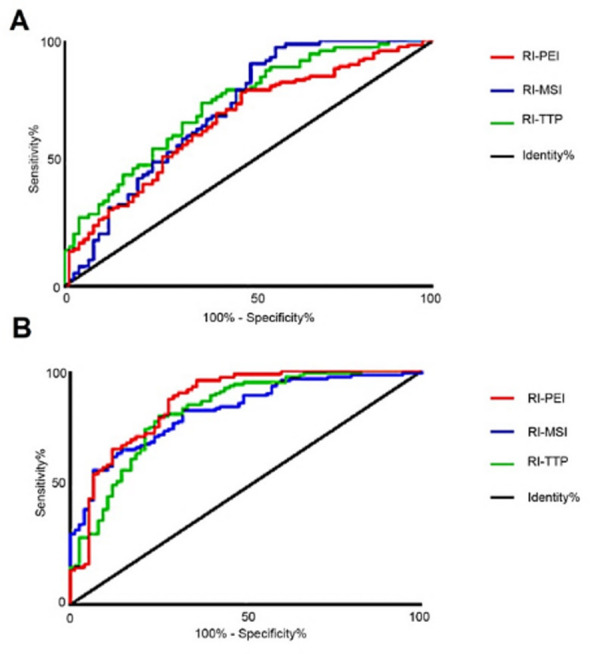
ROC curve analysis of CEUS combined with MRI for diagnosing A) HLN and LNTB; B) LNTB and MLNs.

Initially, we obtained a preliminary diagnosis of the patients in the three groups by using the results of CEUS; next, we applied the results of CEUS and MRI to the diagnoses. Our results showed that the diagnosis accuracy of the combined diagnosis was significantly higher than that of CEUS alone (*P*<0.05; Table-III, and Fig.6A).

**Table-III:** Comparison of diagnostic rates of three groups under different detection methods.

Detection methods		Group-A (n=45)	Group-B (n=55)	Group-C (n=50)
CEUS	Confirmed cases (n)	31	40	37
	Diagnosis rate (%)	68.88^a^	72.73^a^	74.00^a^
Combined method	Confirmed cases (n)	43	52	49
	Diagnosis rate (%)	95.56	94.55	98.00

***Note:***

a P<0.05, compared with combined method.

ROC curve analysis of RI-PEI, RI-MSI, and RI-TTP showed high specificity and sensitivity for the diagnosis and differentiation of HLN and LNTB (*P*<0.05). The specificities and sensitivities of RI-PEI were 63.15% for HLNs and 76.48% for LNTB, with area under the curve (AUC) =0.617; those of RI-MSI were 64.49% for HLNs and 78.82% for LNTB, respectively, with AUC=0.635; and those of RI-TTP were 66.73% for HLNs and 80.12% for LNTB, respectively, with AUC=0.652.

In addition, ROC curve analysis of RI-PEI, RI-MSI, and RI-TTP showed high specificities and sensitivities (*P*<0.05) for the diagnosis and differentiation of LNTB and MLN (Table-IV and Fig.6B). The specificities and sensitivities of RI-PEI were 72.35% for LNTB and 85.24% for MLN, respectively, with AUC=0.749; those of RI-MSI were 71.56% for LNTB and 80.47% for MLN, respectively, with AUC=0.715; and those for RI-DTI were 68.49% for LNTB and 80.13% for MLN, respectively, with AUC=0.708.

## DISCUSSION

We conducted CEUS combined with MRI in 150 patients with neck LN-related diseases in our hospital to improve the differential diagnosis pathway for HLN, LNTB, and MLN. Our results showed that CEUS combined with MRI may significantly improve the diagnostic accuracy of HLN, LNTB, and MLN.

CEUS effectively improves the sensitivity and specificity of ultrasound diagnoses by enhancing the scattering echo.^10^ CEUS diagnoses are made on the basis of image, blood perfusion and abnormal echo results of organs or target tissues. The procedure is non-toxic, has strong scattering, small in diameter, and high stability. It has been widely used in the clinical diagnosis of a variety of diseases.^11^ MRI is a relatively new clinical medical imaging technology available since the 1990s. It mainly uses a static magnetic and radio-frequency magnetic fields to image human tissues displaying diseased organs and early pathological changes from the inside.^12^ In recent years, the application of MRI in medicine has gradually increased, especially in cardiovascular, cerebrovascular, thoracic, and abdominal organs, tumors and other diseases.^13^

CEUS and MRI are the main imaging methods used to differentiate benign and malignant cervical lymph nodes.^14^ In this study, the blood flow types were predominantly of mixed type in Group-A, of lymphatic hilum type in Group-B, and of marginal type in Group-C. MLNs have a damaged lymphoid tissue cellular structure due to the massive invasion by malignant tissues, their blood flow type becomes mixed, and cervical lymph node junction is mainly of the lymphatic hilum type.^15^ The blood flow RI level may be affected because enlarged lymph nodes in the neck may have damaged blood vessels, which may cause vascular stenosis and increases RI values. However, excessive proliferation of malignant tissues in cervical lymph nodes cause damage to the normal boundary and compress blood vessels, resulting in short circuits of dynamic and static veins, and eventually leading to further RI value increases.^16^ Choi et al. has demonstrated that the mean RI in LNs involved with metastases was much higher in LNs affected by benign processes.^17^ Similarly, in this study, we found that the RI values in Group-C were significantly higher than those in Groups-A and B.

Studies have reported the high sensitivity and specificity of MRI in detecting lymph nodal metastases^18^,^19^ PEI, MSI, and TTP are semi-quantitative parameters of MRI. Positive enhancement integral (PEI) shows the difference in perfusion signal intensity before and after lesion enhancement. MSI reflects blood flow in organ microcirculation. TTP describes the time for a contrast agent to reach its maximum concentration.^20^ In the present study, MRI PEI and MSI values were highest in Group-C, followed by those in Group-A. The TTP values in Group-B were the highest, followed by those in Group-A. It is basically in line with Yu et al as benign LN perfusion enhancement time is longer, with low clearance speed compared with LN metastasis.^21^ This is probably related to the massive proliferation of cells in malignant LNs that damage the lymphatic hilum, which leads to an arteriovenous short circuit, and changes the relevant perfusion parameters.^22^

Our results showed that the diagnostic accuracy of the combined approach was significantly higher than that of CEUS alone, which is consistent with the findings by Wendl et al.^23^ Improving the diagnostic pathway of cervical LN diseases is the prerequisite for determining their clinical treatment. Combining CEUS with MRI significantly increases the diagnostic accuracy of HLN, LNTB, and MLN, which is a fundamental step for improving treatment outcomes. In addition, the ROC curves showed high specificities and sensitivities of RI-PEI, RI-MSI, RI-TTP levels for differentiating HLN from LNTB, and LNTB from MLN. Thus, CEUS and MRI can be combined to improve the diagnostic success rate for cervical LN diseases, thereby promoting prompt treatments.

### Limitations of the study:

It is a single-center retrospective study, future multicenter randomized controlled studies are required to further validate our findings. Furthermore, we only investigated HLN, LNTB or MLN in this study, other rare cervical lymph node diseases were not studied.

## CONCLUSION

CEUS combined with MRI can significantly facilitate the differential diagnosis for HLN, LNTB, and MLN. The two diagnosis methods can be combined to improve the diagnosis accuracy of cervical lymph node diseases and promote prompt treatments.

### Authors’ contributions:

**XY** conceived and designed the study.

**WZ, NH, DS and YZ** collected the data and performed the analysis.

**XY** was involved in the writing of the manuscript and is responsible for the integrity of the study.

All authors have read and approved the final manuscript.`
